# Improving Well-being and Health for People with Dementia (WHELD): study protocol for a randomised controlled trial

**DOI:** 10.1186/1745-6215-15-284

**Published:** 2014-07-12

**Authors:** Rhiannon Whitaker, Jane Fossey, Clive Ballard, Martin Orrell, Esme Moniz-Cook, Robert T Woods, Joanna Murray, Jane Stafford, Martin Knapp, Renee Romeo, Barbara Woodward Carlton, Ingelin Testad, Zunera Khan

**Affiliations:** 1 North Wales Organisation for Randomised Trials in Health, Bangor University, Holyhead Road, Bangor, Gwynedd LL57 2PZ, UK; 2 Psychological Services, Oxford Health NHS Foundation Trust, Fulbrook Centre, Oxford OX3 7JU, UK; 3 Wolfson Centre for Age Related Diseases, King’s College London, Guy’s Campus, London SE1 1UL, UK; 4 University College London, 67-73 Riding House Street, London W1W 7EJ, UK; 5 Institute of Rehabilitation: Dementia Applied Research Centre, University of Hull, Health House, Grange Park Lane, Hull, East Yorkshire HU10 6DT, UK; 6 Dementia Services Development Centre, Wales, Institute of Medical and Social Care Research, Bangor University, Holyhead Road, Bangor, Gwynedd LL57 2PX, UK; 7 Section of Mental Health and Ageing, Health Service and Population Research Department, The Institute of Psychiatry at King’s College London, PO26, The David Goldberg Centre, De Crespigny Park, London SE5 8AF, UK; 8 London School of Economics and Political Science, Houghton Street, London WC2A 2AE, UK; 9 Alzheimer’s Society, Devon House, 58 St Katharine’s Way, London E1W 1LB, UK

**Keywords:** Dementia care homes, Quality of life, Antipsychotic medication, Behavioural symptoms, Cost effectiveness, Implementation, Person-centred care, Social interaction

## Abstract

**Background:**

People with dementia living in care homes often have complex mental health problems, disabilities and social needs. Providing more comprehensive training for staff working in care home environments is a high national priority. It is important that this training is evidence based and delivers improvement for people with dementia residing in these environments. Well-being and Health for People with Dementia (WHELD) combines the most effective elements of existing approaches to develop a comprehensive but practical staff training intervention. This optimised intervention is based on a factorial study and qualitative evaluation, to combine: training on person-centred care, promoting person-centred activities and interactions, and providing care home staff and general practitioners with updated knowledge regarding the optimal use of psychotropic medications for persons with dementia in care homes.

**Design:**

The trial will be a randomised controlled two-arm cluster single blind trial that will take place for nine months across 80 care homes in the United Kingdom.

**Discussion:**

The overarching goal of this trial is to determine whether this optimised WHELD intervention is more effective in improving the quality of life and mental health than the usual care provided to people with dementia living in nursing homes. This study will be the largest and best powered randomised controlled trial (RCT) evaluating the benefits of an augmented person-centred care training intervention in care homes worldwide.

**Trial registration:**

Current controlled trials ISRCTN62237498

Date registered: 5 September 2013

## Background

There are more than 750,000 people with dementia in the United Kingdom, at least 250,000 of whom live in care homes [1]. These are the individuals with the most complex needs, often with cognitive and functional impairments in combination with significant behavioural and psychological symptoms [2,3]. The main staff members providing the frontline care often have minimal formal training, yet are being asked to undertake an extremely challenging role which requires a high degree of skill. One of the consequences of this at a national level has been the widespread use of antipsychotic medication for people with dementia, with widespread adverse consequences including a significant increase in mortality [2,3].

A high priority task is to provide more comprehensive training for staff working in care home environments, but it is important that this training be evidence based and deliver improvement for people with dementia residing in these environments [4]. Several studies have been undertaken evaluating person-centred care (PCC) training as an intervention in cluster randomised controlled trials (RCTs) or intervention studies with a quasi-experimental design. A meta-analysis across these studies has indicated that PCC training does deliver some significant benefits, including a reduction in antipsychotic use and an improvement in symptoms of agitation [4,5]. However, benefits are inconsistent across individual studies and, perhaps most importantly, there is no clear evidence of an improvement in quality of life. Whilst PCC training is of some value, there is a clear need to optimise these interventions.

To take this work forward, the National Institute of Health Research (NIHR) funding the Well-being and Health for People with Dementia (WHELD) programme undertook a factorial study to determine whether social intervention, exercise and formal antipsychotic review added significant benefit to PCC training. Based on the results, an optimised intervention has been developed combining PCC training, social intervention and a modified approach to antipsychotic review. The current study will examine this optimised intervention in a parallel group cluster RCT comparing the intervention to usual care over nine months.

### Rationale for current study

There is strong evidence that a number of interventions confer some benefit, but no single intervention has achieved both an improvement of mental health and a reduction of antipsychotic use in people with dementia; and none of the interventions conferred a direct benefit to the quality of life (QoL) of people with dementia in care homes.

The overarching goal of this programme is to determine whether this optimised WHELD intervention, combining PCC, promoting person-centred activities and interactions and providing care home staff and general practitioners with updated knowledge regarding the optimal use of psychotropic medications for persons with dementia in care homes, is more effective in improving the QoL and mental health than usual care for people with dementia living in nursing homes. This optimised WHELD intervention is based on the findings of the factorial study [6] and qualitative evaluation designed to facilitate the design of the current study; a multicentre, two-arm cluster RCT, which is designed to establish the value of the WHELD intervention.

### Study objectives

The primary objective is to determine whether the optimised WHELD intervention will significantly improve QoL for people with dementia in comparison to the usual care provided in care homes.

Evaluations will be undertaken to understand the breadth of additional benefits conferred by the interventions compared with treatment as usual.

### Secondary objectives

Key secondary objectives will be to determine the specific impact of the optimised WHELD intervention on a range of outcomes including mental health and unmet needs, use of psychotropic drugs, pain, the quality of the interaction of care staff with people with dementia, and a person-centred environment in care home settings and to overall provide a cost-effective, simple and practical intervention.

### Hypotheses

We hypothesise that the intervention will significantly improve several key outcomes. Specifically, we hypothesise that, compared to treatment as usual, the optimised WHELD intervention will:

• Improve the QoL for people with dementia living in care homes

The secondary hypotheses are that the optimised WHELD intervention will:

• Reduce the rate of global deterioration

• Reduce agitation and other behavioural and neuropsychiatric symptoms

• Improve mood and depression

• Reduce the use of antipsychotic and other psychotropic drugs

• Reduce pain

• Reduce unmet needs

• Reduce mortality

• Provide a cost-effective intervention

• Improve the quality of interactions between staff and residents

• Provide a person-centred environment in care home settings

## Methods/design

### Overall design

The study design is a cluster randomised controlled, two-arm trial to be run in up to 80 care homes. It is estimated that each cluster will include a minimum of 12 participants. Each cluster in the optimised WHELD intervention arm will receive the intervention for nine months. Evaluations will be undertaken to understand the breadth of benefits conferred by the intervention to be assessed when used in comparison with the progress of the participants residing in the care homes allocated to the treatment as usual (TAU) trial arm.

### The WHELD intervention

The optimised WHELD intervention consists of a combination of elements taken from the interventions trialled in our previous factorial study [6] to form a single formulated, manualised intervention. The optimised WHELD intervention training will focus on PCC, promoting tailored person-centred activities and interactions, and will provide care home staff and general practitioners with updated knowledge regarding the optimal use and monitoring of psychotropic medications for people with dementia in care homes.

The WHELD package will consist of a PCC intervention, which primarily uses the tools developed in evidence-based approaches for improving care in care homes, which was shown to be effective in previous RCTs [7-9]. Additional supplementary materials have been drawn from the best available training manuals from a robust review of available materials [10-12] conducted as part of the WHELD study. The feasibility of incrementally combining these materials was evaluated in our previous factorial study [6], and the findings have been used to create the current optimised intervention.

The PCC intervention has five foci:

• Embedding an understanding of dementia and PCC

• Developing the staff’s understanding about the relationship between an individual’s experience and that individual’s behaviour and well-being

• Having care homes embed processes for self-assessing how their practices deliver PCC

• Enabling staff to recognise the impact of staff - resident interactions on the care experience

• Having staff and residents implement PCC planning and individualised care practice based on these principles

This intervention will use evidence-based approaches and specific communication skills training to enhance staff-resident interactions. The intervention will also incorporate evidence-based approaches to encourage person-centred activities and interaction.

The intervention will be delivered by trained research therapists, who will receive an intensive ten-day training package, each of whom will coordinate the delivery of the intervention in the care homes, supported as appropriate by senior staff at each centre. Part of the intervention will involve the training of two lead care staff members (WHELD Champions) in each care home over a period of four months (one training day per month) with additional coaching and supervision over the nine-month period, to support their implementation of the intervention, The intervention manual and materials will be made available following publication of trial results and will also be submitted for publication in accordance with the trial internal and external dissemination plan.

### Control arm

The control group will receive TAU. Care homes in the control group will need to demonstrate a minimum acceptable standard of care, and this information will be collected as part of the quality screening at the selection stage.

An inventory of the standard of care of control arm homes will be undertaken, in order to be able to describe the features of the homes and to document the training they access during the course of the intervention delivery period.

Monthly contact with control group care homes will be maintained during the programme, to ensure that Serious Adverse Event (SAE) reporting occurs.

### Design and Consolidated Standards of Reporting Trials (CONSORT) diagram

**Figure 1:**
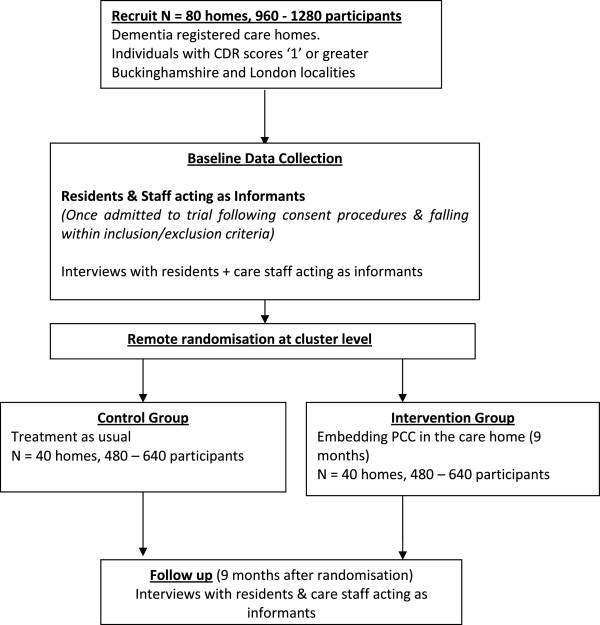
Design and CONSORT diagram.

**Figure 2:**
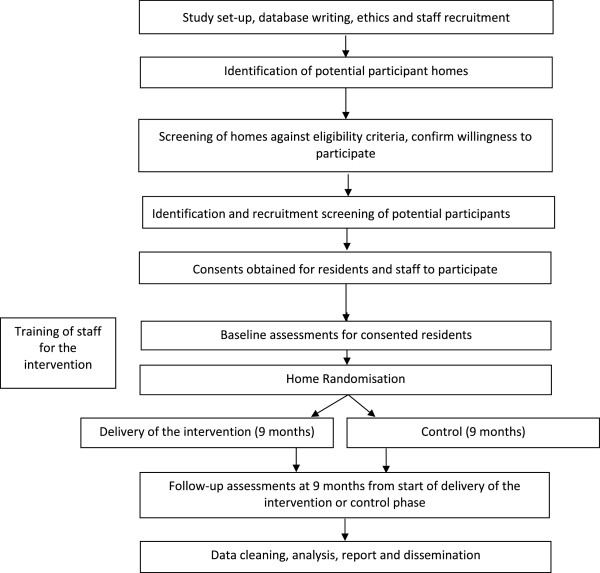
Flow chart of WHELD programme milestones.

This will be a pragmatic cluster RCT testing the experimental intervention with a sample of residents with dementia in care homes. The sample will be cluster-randomised by care home, with all recruited residents in a given care home being allocated to either the experimental or control arm. Figure 1 (the design and CONSORT diagram) shows the stages of the trial. Figure 2 shows the programme milestones.

### Number of participants and power of the study

Up to 80 suitable care homes, or units within larger homes, will be identified, recruited and randomised, and the minimum target participant recruitment is 12 individuals with dementia per care home. Therefore, the target minimum sample size is 960, with a suggested upper recruitment limit of approximately 1,280 (that is, 16 individuals with dementia per care home). There will be no more than two units in any care home.

Baseline and follow-up data will be collected on all consented residents who meet the inclusion criteria at each participating care home. Taking into account the likely loss to follow up, it is estimated that, on average, 8 of the 12 will be followed up at nine months, giving a total sample size of 640. This trial is cluster randomised to take account of the fact that the participants live together in care homes. Re-analysis of data from previous studies suggests that intra-home correlation coefficients rarely exceed 0.05. As the ‘variance inflation factor’ (VIF) of this cluster randomised trial [1 + (8 - 1) × 0.05] = 1.35, the likely useable sample size of 640 participants will give 90% power using a significance level of 5% to detect a standardised effect size of 0.3 SDs, which is usually taken as the lowest threshold of a clinically meaningful benefit [13].

The observed SD for our population on DEMQOL taken from our pilot study is approximately 12. The standard error of measurement (SEM), derived by multiplying the SD of the sample by the square root of one minus its reliability coefficient, has been described as a useful statistic for assessing individual change on health-related QoL instruments [14]. Smith *et al.*[15] reported DEMQOL’s reliability coefficient as 0.82. Therefore, the SEM for this data is 12*sqrt (1-.82) = 5.1. This provides further support that we are measuring clinically meaningful change in DEMQOL [16]. Expressing this change as an effect size gives 5.1/12 = 0.425.

### Randomisation

Care homes will be allocated to either receive the optimised WHELD intervention or TAU using secure web access to the remote randomisation centre at North Wales Organisation for Randomised Trials in Health Clinical Trial Unit (NWORTH CTU), at Bangor University. This system will be set up, maintained and monitored independently of the trial statistician or other trial staff. The randomisation will be performed by dynamic allocation [17] to protect against subversion while ensuring that the trial maintains good balance to the allocation ratio of 1:1 both within each stratification variable and across the trial. Care homes will be stratified by region and size. The system has been coded and validated in the R statistical package.

### Outcome and study eligibility measures

All outcomes will be assessed prior to randomisation and after nine months of the intervention. Outcome measures will consist of questionnaires and direct observations, using standardised, evidence-based scales and other information obtained from an informant (the key worker or another member of the staff who knows the participant well). Participant and care home demographics will be collected and measures taken as described in the following paragraphs.

The DEMQOL [15] assesses the health-related quality of life (HRQoL) for people with dementia. The measure consists of two questionnaires. DEMQOL, conducted with a person with dementia, is a 28-item interviewer-administered questionnaire with a score range of 28 to 112. DEMQOL-Proxy is a 31-item interviewer-administered questionnaire answered by a caregiver with a score range of 31 to 124. The DEMQOL-Proxy is the primary outcome measure for this study. The measure is also validated as a method for calculating quality-adjusted life-year (QALY) for health economic analysis.

The Quality of Life in Late-Stage Dementia (QUALID) scale [18] is a reliable and valid scale, administered to caregivers, for rating quality of life in people with late-stage dementia. The scale measures 11 observable behaviours indicating activity and emotional states. Ratings are made for observations made over the preceding seven days. Items are rated on a five-point Likert scale with QUALID scores ranging from 12 to 45 points; lower scores reflect a higher quality of life (QoL).

The Clinical Dementia Rating (CDR) [19] is a validated scale used to quantify the severity of symptoms of dementia. Using a structured interview, six domains are assessed in terms of a person’s cognitive and functional performance. These include memory, orientation, judgment and problem solving, community affairs, home and hobbies, and personal care. CDR ratings are 0 for healthy people, 0.5 for questionable dementia and 1, 2 and 3 for mild, moderate and severe dementia. Scores in each of these domains are combined to obtain a composite score ranging from 0 (none) through 3 (severe).

The Functional Assessment Staging Tool (FAST) is a validated functional assessment scale of elderly people with dementia [20]. The FAST is an ordinal scale ranging from 1 (indicating normal function) to 7 (indicating severe dementia). Levels 6 and 7 are divided into specific subscales yielding 16 possible ratings. Each level is indicated by a functional description with adequate detail for clinical scoring. The FAST score is derived from a caregiver interview.

The Global Deterioration Scale (GDS) [21] is a staging scale indicating deterioration in dementia. The scale details clinical descriptions of seven major distinguishable stages, ranging from normal cognition to severe dementia. Stages 1 through 3 are the pre-dementia stages, While stages 4 through 7 reflect the stages of dementia. People with clinical levels beginning with stage 5 are no longer able to survive without assistance.

Agitation will be assessed using the Cohen-Mansfield Agitation Inventory (CMAI) [22], which is a caregiver’s rating questionnaire to specify agitated behaviour. The CMAI consists of 29 items related to agitated behaviour, each of which is rated on a seven-point scale of frequency, from 1 = never to 7 = several times an hour. The rating is based on a face-to-face interview with a caregiver.

Other behavioural and neuropsychiatric symptoms will be recorded using the Neuropsychiatric Inventory Nursing Home version (NPI-NH). The NPI is a validated structured interview assessment with the informant (care staff), that assesses behavioural disturbances in patients with dementia [23]. This 12-item version consists of ten behavioural and two neurovegetative areas. It provides both a total score as well as scores for a number of subscales (delusions, hallucinations, agitation/aggression, depression/dysphoria, anxiety, disinhibition, elation/euphoria, apathy/indifference, irritability, aberrant motor activity, sleep, and appetite/eating disorders). The frequency, severity and caregiver distress levels for each domain are measured. The total possible maximum score is 144. A higher score reflects increased frequency and severity of the disturbances. This specific version is developed for use in nursing homes, with adapted questions in the standardised interview, and the caregiver distress assessment is adapted to occupational disruptiveness.

Depression will be assessed using the Cornell Scale for Depression in Dementia (CSDD). The CSDD [24] is an assessment of signs and symptoms of major depression in patients with dementia. The CSDD uses a comprehensive interviewing approach that derives information via semi-structured interviews with the participant and the care staff. Many of the items to be completed during the patient interview can be filled in after direct observation of the patient. The final ratings of the CSDD items represent the rater's clinical impression rather than the responses of the informant or the patient. Each item is rated for severity on a scale of 0 to 2 (0 = absent, 1 = mild or intermittent, 2 = severe), and the item scores are then added. Scores above 10 indicate a probable major depression. Scores above 18 indicate a definite major depression. Scores below 6 as a rule are associated with absence of significant depressive symptoms.

Information on antipsychotic use (number of people taking antipsychotics and dose) will be taken from participants’ drug charts, as will the use of other psychotropic (sedative) drugs and other medication.

The Abbey Pain Scale [25] is an observational brief indicator of pain for people with end-stage dementia. The scale is rated on six non-verbal indicators of pain, where 0 is none and 3 is severe.

The Health Questionnaire (Proxy version EQ-5D-5 L), which is based on the EQ-5D-3 L [26] is used to enable an additional health economic evaluation. The questionnaire is short and brief, but importantly will provide greater comparability with other studies.

The Camberwell Assessment of Need for the Elderly (CANE Version IV) [27] is a comprehensive assessment assessing 24 areas of social, medical, psychological and environmental needs. The overall rating on the CANE will be based on all the information gathered throughout the structured interview with the care staff and on the information collected through looking at the case-note reviews and observations within the care home. We will complete this instrument only after the nine months intervention time point.

We will use the Client Service Receipt Inventory (CSRI) [28] to estimate the cost of service packages for each participant in the study. Information is collected on the current living arrangements, and use of hospital, community-based and day services over a defined retrospective of three months in this study. The data collected through the CSRI will be used to calculate service costs and total costs of care.

The Quality of Interaction Schedule (QUIS) [29] is an observational tool which measures the quality of interactions between staff and resident and person-centred environment in care home settings.

The Enhancing the Healing Environment (EHE) Environmental Assessment Tool [30] is a comprehensive observational assessment of the care environment. Assessors can carry out the assessment primarily by walking through the home and directly observing the building and the way it is used in practice. The EHE Environmental Assessment Tool has been produced by the Department of Health and King’s Fund as part of a national survey; therefore, using this tool in our study will provide benchmarking against national data.

### Intervention fidelity

To monitor intervention fidelity, each therapist will be required to maintain a detailed log of the type and frequency of interventions and an audit of detailed intervention plans describing each intervention undertaken, audit of care plans and a supervision log.

### Economic evaluation

Comprehensive data on health and social care service use and medications used by participants in the study will be collected using a tailored version of the CSRI. We will also collect data on staff inputs and support received by the study participants as part of the interventions.

Services and staff inputs will be costed as long-run marginal opportunity costs (LRMCs) using national figures. Where national figures are not available or not suitable, we will calculate best estimates of LRMC values from locally collected expenditure figures. Data on intervention costs and service costs will be examined alongside data on the main outcomes in a series of cost-effectiveness analyses. Each cost-effectiveness analysis will be conducted from the perspective of the NHS and social services.

Quality-adjusted life-year (QALY) gains generated from the DEMQOL will be used in turn in a series of cost-effectiveness analyses. The evaluation will include the plotting of cost effectiveness acceptability curves generated from bootstrap analyses. Sensitivity analyses will explore the impact of changes in the findings in key costs and outcome assumptions.

### Care home selection: inclusion and exclusion criteria

Care homes with a high proportion of residents thought to have dementia will be identified, screened and recruited to the study as described below.

An initial search for suitable care homes will be made using local care home directories (this will include care homes that are part of care home research networks). These care homes will be screened for the following inclusion/exclusion criteria.

Inclusion criteria:

• Care homes that identify themselves as catering for people with dementia within their literature

• Care homes that are able to demonstrate a minimum acceptable standard of care according to the Care Quality Commission (CQC)

Exclusion criteria:

• Less than 60% of the residents have dementia

• Care home is receiving special support from their local authority

• Care home has failed to meet the five CQC care home quality standards checks with more than one black mark (at least one standard in this area was not being met and improvements are required) or one red mark (at least one standard in this area was not being met and enforcement has been taken):

1. Standards of treating people with respect and involving them in their care

2. Standards of providing care, treatment and support which meets people’s needs

3. Standards of caring for people safely and protecting them from harm

4. Standards of staffing

5. Standards of management

The research teams in each study site will contact all care homes meeting the inclusion/exclusion criteria on the list by email, mail and/or phone call. The aims of this exercise will be to:

• Find out whether the care home might be interested in participating in the study; and

• Screen care homes further against secondary exclusion criteria to determine their suitability.

Secondary exclusion criteria:

• Insufficient staffing resource. Care home unable to provide care staff champions or staff able to act as informants for participant assessments

• Anticipated major change. Anticipated events or major changes anticipated to take place in the next 12 months, which might impact involvement in the research

• Involvement in other research. Care home involved in other research projects, which might affect their suitability to take part in this study or place too much burden on staff and residents

• Undergoing systematic programme of service improvement: Care home involved in a systematic programme of service improvement (such as intensive Dementia Care Mapping).

Once a care home meets the inclusion/exclusion criteria and the manager has given consent for the study to take place, the identifying, consenting and baseline assessments will commence. Once this process has been completed, the researcher will randomise the care home using NWORTH’s online randomisation system. Then the study site will initiate the main trial procedures at the care home.

Care home withdrawal from the study will be considered on an individual basis with the home in the event circumstances change following the consent procedures.

### Recruiting centres

Three recruiting hubs based in London and Buckinghamshire will each aim to recruit between 20 and 25 care homes.

### Participant selection: inclusion, exclusion and withdrawal criteria

All residents who would be potentially eligible for evaluation will be identified by the care home staff.

Inclusion for evaluation:

• All individuals residing in participating care homes who meet diagnostic criteria for dementia and/or score ‘1’ or greater on the CDR [19].

Exclusion from evaluation:

• Any resident from whom consent or the advice of a consultee cannot be obtained.

Withdrawal criteria:

• Individual participants will be able to withdraw from the study evaluation at any time.

### Data management and analysis

#### Safety reports of serious adverse events (SAEs)

Reports of SAEs will be performed according to the National Research Ethics Service (NRES) and NHS guidelines for safety reporting for research other than Clinical Trial of Investigational Medicinal Products (CTIMP).

### Assessment and follow-up

Assessments will be made at pre-baseline to assess the suitability of the care homes for inclusion. This process will include assessments to identify the total number of participants likely to be eligible for screening. Participants will be screened and consent obtained prior to baselining and to the care homes being randomised. Follow-up assessments will be made at nine months after commencement of the intervention.

### Data management and analysis

It is planned that anonymous data and all appropriate documentation will be kept securely for a period of seven years following the completion of the trial, subject to discussion with relevant ethics committees.

### Quantitative data management

Administrative databases will be held at the study centre. All participants and care homes will be identified by a unique study number; this number will be used to tag all research data sent outside the study centre, for example to NWORTH CTU. Quantitative research data will be entered via a web interface to the MACRO™ research databases held at NWORTH. The research team in the study centre will conduct primary data management, and the secondary cleaning and preparation of the data for analysis will be conducted by NWORTH. Consideration will be given to the differential time between baseline assessments and the start of intervention, adjusting for the time differences to an analysis as necessary.

### Quantitative analysis

Outcome measures for the study will be assessed at baseline and at nine months. All the outcome measures collected will be described and reported using appropriate descriptive statistics and tabular and graphical techniques. Means with 95% confidence intervals will be quoted and a 5% significance level will be reported.

The Consolidated Standards of Reporting Trials (CONSORT) diagram information will be presented in order to identify any differential dropout between the arms of the trial.

The analysis of the quantitative outcomes will involve a multilevel analysis of covariance (ANCOVA). Logistic regression will be used to assess whether the missing status can be predicted from any of the factors and covariates measured at baseline. The variables which are identified to affect the missing status will be included in the analysis model [31]. The primary outcome measure (DEMQOL proxy) [22] and the secondary outcome measures will be analysed using the multilevel modelling approach to ANCOVA. The value at nine months will be the response. The baseline value will be the covariate. The key factor will be group (treatment or control). The multilevel nature of the design will be represented by two levels: care home and individual residents in the care home.

Other covariates, whether at the care home level (for example, number of residents) or the resident level (age, gender) will be investigated to see whether they have an effect on the response measure and included as necessary.

A full statistical analysis plan will be developed through the course of the study.

### Regulatory and management issues

#### Ethical approval

Ethical approval was successfully obtained from the National Research Ethics Service (NRES) Committee South Central - Oxford C via a central ethics application for the research trial (Rec reference: 13/SC/0281, Protocol number: WHELD WP5, IRAS project ID: 128232). Site Specific Assessments (SSAs) were completed at Guys and St Thomas’ Hospital, Oxford Health NHS Foundation Trust and North East London NHS Foundation Trust and approved prior to accepting participants into the study. The study will be conducted in accordance with the recommendations for physicians involved in research on human subjects adopted by the 18th World Medical Assembly, in Helsinki 1964, and later revisions.

The study may be subject to inspection and audit by the sponsors and other regulatory bodies to ensure adherence to Good Clinical Practice (GCP) and the current NHS Research Governance Framework for Health and Social Care.

#### Consent

Care home managers of the participating care homes will be provided with the inclusion criteria and asked to identify potential participants.

All of the participants will be people with dementia living in the participating care homes. It is therefore unlikely that more than a very small number of potential participants will have the capacity to provide informed consent. The care home managers will therefore then ask the consultee if they are happy to be contacted by the research team. Initially, this will be undertaken through a letter to the consultee and might be followed by personal conversation, as appropriate. If permission is granted, the research team will discuss the study in further detail with the consultee via telephone conversations or a meeting, depending on the preference of the individual. In addition, the relevant information sheet will be given or posted to the consultee for their information prior to signing the declaration form.

If the personal consultee feels that it is appropriate for the person to take part, but feels they potentially have the capacity to make their own decision, an assessment of capacity will be undertaken by a study clinician. If the individual does have capacity, a simplified written information sheet will be provided to the individual and the study will be explained to the person, by a member of the research team with appropriate training and skills, and where possible with the next of kin also present. If the individual wishes to take part, written consent will be taken. In addition, as a measure of good practice, signed assent will be requested from the consultee.

For individuals who do not have capacity, an appropriate member of the research team will discuss the study in further detail with a consultee of the potential study participant through telephone conversations or a meeting, depending on their preference. No individuals will participate in the evaluation without signed, written consent from themselves or a signed declaration from their consultee. All participants are free to withdraw from the study at any time without giving reasons and without prejudicing further treatment. For people who lose capacity, the consultee will be approached and the issue of on-going participation will be discussed. If the consultee wants the person to continue to participate, they will be asked to sign the declaration form. Ongoing participation would just require the completion of the nine-month outcome assessment.

### Confidentiality

The Chief Investigator will preserve the confidentiality of participants taking part in the study and is registered under the Data Protection Act (DPA, 1998). The research will follow DPA guidance. Only members of the research team will have access to the original data, which will be stored in a locked filing cabinet. Participants’ personal details will be stored separately from the original data, and will be kept in a separate file on a password protected computer at the study centre. Each participant will be assigned an identification code, which will be used in all data storage files; these files will not contain names or any other means of personal identification. All personal details will be deleted on completion of the study.

### Indemnity

King’s College London will be responsible for providing indemnity for negligent liability. No provision is made for non-negligent liability, which will be covered by the usual procedures by NHS or the care provider as applicable.

### Sponsor

King’s College London will act as the sponsor for this study.

### Funding

The National Institute of Health Research (NIHR) Programme Grants for Applied Research are funding this study. No per participant or per care home payments are being made as part of this study.

### Study management

This study will be managed by the Programme Management Group (PMG), as part of the overall WHELD programme. The PMG will involve all the Principal Investigators, the Programme/Trial Manager and a Consumer Representative. The group will meet at three-month intervals and additionally as required. Part of the remit of the PMG will be to oversee overall progress of the programme and monitor progress against milestones. Any discrepancy from milestones will be highlighted and a plan, developed to address the difficulties, will be instigated. For the purposes of this trial, the PMG will be acting as the Trial Management Group (TMG), with direct oversight of and responsibility for this study. The Programme/Trial Manager will send a written report to the chairman of the PMG before each TMG meeting, detailing progress.

The Programme Steering Committee (PSC) will be responsible for overall WHELD programme governance and reporting to the sponsor. An independent subcommittee of the PSC will act as the Data Monitoring and Ethics Committee (DMEC) for the study. Both committees will meet annually and will receive annual reports from the Programme Manager and the Trial Statistician. The DMEC and PSC will monitor recruitment, ethical issues and safety and will have the ultimate responsibility for the continuation or discontinuation of the study. The DMEC will have an Independent Clinician as chair, a further Clinician and a Statistician. The PSC will consist of six members, including a Consumer Representative. Therefore, for the purpose of this trial, the PSC will be responsible for trial governance, reporting to the sponsor.

The day-to-day management of the trial will be conducted by the Programme/Trial Manager, who will report to the Chief Investigator on a regular basis. Any non-urgent major decisions will be made by the PMG.

## Discussion

This study will be the largest and best powered RCT evaluating the benefits of an augmented PCC training intervention in care homes worldwide and will be the first trial that is adequately powered to determine whether this intervention will improve QoL. The Health Economic component will provide the first comprehensive evaluation of cost effectiveness in this type of study.

## Trial status

The trial is in the recruiting phase at the time of manuscript submission.

## Abbreviations

ANCOVA: analysis of covariance; CANE: Camberwell Assessment of Need for the Elderly; CDR: Clinical Dementia Rating; CI: Chief Investigator; CMAI: Cohen-Mansfield Agitation Inventory; CONSORT: Consolidated Standards of Reporting Trials; COREC: Central Office for Research Ethics Committees; CQC: Care Quality Commission; CSDD: Cornell Scale for Depression in Dementia; CSRI: Client Service Receipt Inventory; CTIMP: Clinical Trial of Investigational Medicinal Products; CTU: Clinical Trial Unit; DEMQOL: Measure of Health-Related Quality of Life for People with Dementia; DMEC: Data Monitoring and Ethics Committee; DPA: Data Protection Act; EHE: Enhancing the Healing Environment; FAST: Functional Assessment Staging Tool; GCP: Good Clinical Practice; GDS: Global Deterioration Scale; HRQoL: health-related quality of life; ISRCTN: International Standard Randomised Controlled Trial Number Register; LRMC: long-run marginal cost; NDS: National Dementia Strategy; NIHR: National Institute of Health Research; NPI-NH: Neuropsychiatric Inventory-Nursing Home version; NRES: National Research Ethics Service; NWORTH: North Wales Organisation for Randomised Trials in Health; PCC: person-centred care; PMG: Programme Management Group; PSC: Programme Steering Committee; QALY: quality-adjusted life-year; QoL: quality of life; QUALID: Quality of Life in Late-Stage Dementia; QUIS: Quality of Interaction Schedule; RCT: randomised controlled trial; REC: Research Ethics Committee; SAE: Serious Adverse Event; SD: standard deviation; SSA: Site Specific Assessment; TAU: treatment as usual; TMG: Trial Management Group; WHELD: Well-being and Health for People with Dementia - an optimised intervention ’welding together’ the most effective elements of the best currently available intervention programmes and a standardised manual and training programme.

## Competing interests

The authors declare that they have no competing interests.

## Authors’ contributions

CB is the chief investigator of the WHELD programme. JF and MO are site leads for the trial and, along with MK, EM-C, JM, RTW and BW-C, are principal investigators and were involved in design, grant applications, and protocol development and are members of the programme management group. RhW led the development of the protocol, provides statistical support to the trial and is a member of the programme management group. JS manages the trial on a day-to-day basis; RR supports the economic evaluation at the London School of Economics; IT and ZK are members of the research team at King’s College, London. All authors read and approved the final manuscript.
